# Editorial: The thyroid and Covid-19, volume II

**DOI:** 10.3389/fendo.2023.1331452

**Published:** 2023-11-27

**Authors:** Gabriela Brenta, Marco António Campinho, Celia Regina Nogueira, Jose Sgarbi

**Affiliations:** ^1^ Endocrinology, Dr. César Milstein Care Unit, Buenos Aires, Argentina; ^2^ Faculty of Medicine and Biomedical Sciences, Universidade do Algarve, Faro, Portugal; ^3^ Algarve Biomedical Center-Research Institute, Universidade do Algarve, Faro, Portugal; ^4^ Department of Internal Medicine, Medical School Botucatu, São Paulo State University (UNESP), Botucatu, Brazil; ^5^ Thyroid Unit, Division of Endocrinology and Metabolism, Department of Medicine, Faculdade de Medicina de Maríli, Marilia, Brazil

**Keywords:** COVID - 19, thyroid, pandemic (COVID19), COVID 19 vaccines, thyroid cancer

## More insights into thyroid and COVID-19

Coronaviruses are enveloped RNA viruses of wide distribution in humans associated with mild respiratory disease. By contrast, severe acute respiratory syndrome coronavirus (SARS-CoV) is one of those coronaviruses that can cause fatal illness. In late December 2019, an outburst of pneumonia of unknown cause in Wuhan, China, was identified as the early stage of the coronavirus disease (COVID-19) pandemic outbreak, and the SARS-CoV-2 was found responsible (1).

Two main proteins expressed by SARS-CoV-2 are essential for the manifestations of COVID-19. The first is the transmembrane protease serine 2 (TMPRSS2), which acts on the transcription and replication of the virus. The second is the Spike protein found on the surface of viral particles, which binds to angiotensin-converting enzyme 2 (ACE2) in tissue cells and is a determinant for transmitting infection. Therefore, SARS-CoV-2 infection depends on two steps: ACE2 receptor recognition via Spike protein and cell membrane fusion via transmembrane protease (2).

ACE2 is expressed in different tissues, and the thyroid is no exception (3). It has been shown that the thyroid gland has high expression levels of ACE2, which may explain the direct effects on the thyroid parenchyma, making it more susceptible to viral attack (3).

SARS-CoV-2 infection can lead to thyroid diseases by severely destroying parafollicular and follicular epithelial cells, leading to follicle rupture. As a result, SARS-CoV-2 virus infections are associated with inflammatory thyroid diseases such as subacute thyroiditis, Graves’ disease, thyrotoxicosis, Hashimoto’s thyroiditis, and euthyroid patient syndrome (4). As regards thyroid cancer, the COVID-19 pandemic has also affected its traditional management, and the consequences of this strategic change are largely unknown. Furthermore, it is intriguing that if the virus attacks the thyroid gland, it can also modulate thyroid cancer behavior (5).

In this second volume of COVID-19 and the Thyroid of Frontiers in Endocrinology, nine articles have been included that describe the association between the thyroid gland and the SARS-CoV-2 infection. The topics covered referred to thyroid hormone dysregulation in COVID-19-infected patients and after COVID-19 vaccination, as well as the strategic management of thyroid nodular disease during the pandemic.

In this regard, Silveira et al. described the adapted management of a whole country by retrospectively analyzing the data of thyroid cancer-related procedures in the Brazilian public health system from 2019 to 2021. According to medical guidelines recommendations, there was a considerable reduction in the number of FNABs (29%), oncologic thyroidectomies (17%), and RAI therapies (28%) in 2020. Due to the lack of information about patients’ clinical and oncological features, the long-term consequences of this adopted strategy on thyroid cancer care could not be evaluated. However, since the proportion of thyroidectomies decreased during the pandemic but not significantly, it may be speculated that surgery for high-risk thyroid cancer was prioritized. Therefore, this study may exemplify active surveillance for low-risk thyroid tumors in a large-scale model.

In other terms, the challenging situation of managing respiratory distress due to giant goiter and concomitant severe COVID-19 disease was reported in four patients by Wang et al. In three cases where emergency thyroid surgery was implemented, the respiratory tract obstruction was relieved, and dyspnea improved post-surgically. However, the Authors reckon that anesthesia stimulation may have aggravated the inflammatory response attributable to the viral infection. Given the small size of this study, solid conclusions are difficult to obtain. However, as thyroid surgery was generally avoided during the COVID-19 pandemic, this small case-series study reveals critical details of the pros and cons of emergency thyroid surgery in this exceptional context.

As regards thyroid dysfunction during COVID-19, a systematic review and meta-analysis of the English and Chinese population thyroxine levels carried out by Li et al. found that compared to healthy patients, COVID-19 infection showed decreased TSH and FT3 levels, whereas FT4 was increased. Moreover, these findings also indicate that the severity of the infection was positively correlated with the observed changes in thyroxine levels. These results support the notion that FT3, in particular, could constitute an essential clinical index to understand the impact of COVID-19 infection on thyroid function.

It is particularly interesting if post-COVID-19 patients are more predisposed to develop thyroid autoimmunity. Rossini et al. addressed this question by assessing thyroid function and antibodies in a prospective cohort of 599 COVID-19 survivors. The results were compared to a historical control group from the same institution without thyroid disease. TPOab prevalence was almost double (15.7% vs 7.7%) in the COVID-19 group, raising awareness of the immunogenic potential of this disease upon the thyroid, a gland with a high propensity to autoimmunity.

Among the many thyroid function markers found to be impaired by COVID-19 is thyroglobulin. Swiątkowska-Stodulska et al. assessed thyroglobulin (TG) levels as a marker of possible thyroid destruction in 174 patients hospitalized for COVID-19 and after glucocorticoid treatment. TG levels decreased over time in the whole group and did not differ between the patients with normal and abnormal thyroid function tests. However, it is interesting that the decrease was primarily observed in the subgroup of individuals under glucocorticoid therapy. Although this therapeutical approach is widely used in subacute thyroiditis, its potential use to protect the thyroid gland in the setting of SARS-CoV-2 is entirely original.

Using a cohort of Japanese health workers immunized against COVID-19 with the SARS-CoV-2 BNT162b2 mRNA vaccine, Morita et al. found that this vaccine can disrupt thyroid autoimmunity, leading to Graves´ disease. The 12-month follow-up study found that after two doses of the SARS-CoV-2 BNT162b2 mRNA vaccine, several serum markers of Graves´ disease, most notably anti-TSH receptor antibody, increased in female patients. Moreover, after the third dose, there was also an increase in anti-thyroglobulin antibodies. Although larger cohort epidemiologic studies are required, this evidence argues that clinical surveillance on thyroid status should be considered after immunization against COVID-19 to prevent relapse of new onset of Graves’ disease and allow early identification and clinical management of this condition.

To investigate mood changes and thyroid disorders after COVID-19 vaccination in susceptible populations, Ma et al. performed a retrospective multi-center study in Hashimoto’s thyroiditis patients. The thyroid function tests, thyroid antibodies, CRP levels, and Beck Depression Inventory scores of 2765 patients before and 24 weeks after two doses of inactivated CoronaVac (BBIBP-CorV) vaccines were compared to propensity score matched 1288 non-vaccinated controls. Increased levels of TSH were observed in the vaccinated patients, followed by mood changes. Increased baseline levels of TSH, CRP, and TPOab were predictors of a higher incidence of mood changes. These findings might serve to support the long-debated argument on the relationship between depression and thyroid autoimmunity.

Finally, Rossetti et al. elaborated a comprehensive narrative review of several studies reporting the consequences of COVID-19 on the thyroid, focusing on the mechanistic pathways whereby COVID-19-induced cytokine storm might lead to NTIS and thyrotoxicosis. This review is complemented by the study by Zhang et al. also included in this second volume of the Research Topic on the Thyroid and COVID-19. Data obtained from the ThyroidOmics Consortium served for thyroid function analysis, while data on COVID-19 susceptibility and severity were extracted from the COVID-19 Host Genetics Initiative. It was shown that COVID-19 susceptibility might be a risk factor for hypothyroidism, opening the possibility of adding COVID-19 infection to the list of case-finding for hypothyroidism.

## Author contributions

GB: Writing – original draft, Writing – review & editing. MC: Writing – review & editing. CN: Writing – review & editing. JS: Writing – review & editing.
